# Clinical impact of skin autofluorescence on high-sensitivity troponin T in hypertensive patients

**DOI:** 10.1186/s40885-017-0076-y

**Published:** 2017-10-04

**Authors:** Takashi Hitsumoto

**Affiliations:** Hitsumoto Medical Clinic, 2-7-7, Takezakicyou, Shimonoseki City, Yamaguchi 750-0025 Japan

**Keywords:** Skin autofluorescence, High-sensitivity troponin T, Oxidative stress, Arterial stiffness, Hypertension

## Abstract

**Background:**

Recent studies have reported the importance of high-sensitivity troponin T (hs-cTnT) or skin autofluorescence (AF) as a cardiovascular risk factor. However, little is known about the relationship between these two markers. The aim of this study was to clarify the clinical impact of skin AF on hs-cTnT in hypertensive patients, from the perspective of primary prevention of cardiovascular events.

**Methods:**

In total, 457 outpatients on treatment for hypertension [182 men and 275 women; mean (± SD) age, 67 ± 13 y] and with no history of cardiovascular events were enrolled. Hs-cTnT levels and skin AF were measured using commercial devices, and relationships between hs-cTnT levels and various clinical parameters including skin AF were examined.

**Results:**

Hs-cTnT was detected in 405 (88.6%) patients. Skin AF was significantly higher in patients with detectable hs-cTnT than in those without detectable hs-cTnT [2.6 ± 0.5 arbitrary units (AU) vs. 2.2 ± 0.5 AU, respectively, *p* < 0.001]. In patients with detectable hs-cTnT, there was a significantly positive correlation between skin AF and Log-hs-cTnT (*r* = 0.41, *p* < 0.001). Furthermore, multiple regression analysis revealed that skin AF was the strongest variable associated with Log-hs-cTnT as a subordinate factor (β = 0.30, *p* < 0.001).

**Conclusions:**

The results of this study indicate that skin AF is an important determining factor for hs-cTnT elevation in hypertensive patients with no history of cardiovascular events.

## Background

Troponin T is released from the myocardium into the blood following myocardial injury. In addition, recent clinical and epidemiological studies have demonstrated that the blood concentration of cardiac troponin T [i.e., high-sensitivity cardiac troponin T (hs-cTnT)] can be measured using a highly sensitive assay. Blood levels of hs-cTnT are known to be a useful biomarker for evaluating the pathogenesis of heart failure and subclinical myocardial injury or for the prediction of cardiovascular events [1–4]. Furthermore, some studies have indicated the clinical significance of hs-cTnT in hypertensive patients [5–7].

Advanced glycation end products (AGEs) play an important role in various diseases such as diabetes mellitus, kidney diseases, and cardiovascular disease. Among the methods used to evaluate AGEs, skin autofluorescence (AF) is known to be a simple and reliable marker of AGEs in vivo. In fact, some clinical studies have reported a relationship between skin AF and diabetic complications or cardiovascular disease [8–10]. In addition, some studies have reported clinical significance of skin AF in hypertension [11, 12]. To the best of our knowledge no studies have reported a relationship between skin AF and hs-cTnT in hypertensive patients. Therefore, the present study was conducted to clarify the relationship between hs-cTnT and skin AF in hypertensive patients from the perspective of primary prevention of cardiovascular events.

## Methods

### Study population

This study was conducted at the Hitsumoto Medical Clinic in Shimonoseki City, Japan, between March 2015 and February 2017. The study population consisted of 457 hypertensive patients undergoing anti-hypertensive treatment. No patients in this study had a history of cardiovascular events such as ischemic heart disease, stroke, perivascular disease, or heart failure. There were 182 (39.8%) males and 275 (60.2%) females in this study, with a mean age ± SD of 67 ± 13 y. All participants provided informed consent, and the study protocol conformed to the ethical guidelines of the Declaration of Helsinki. The study was approved by the Local Ethics Committee of the Hitsumoto Medical Clinic (Approval number: 2015–04).

### Measurement of skin AF

Skin AF was measured using a commercial instrument (AGE Reader™; DiagnOptics, Groningen, Netherlands), as previously described [13, 14]. AF was defined as the average light intensity per nanometer between 300 nm and 420 nm. The levels of skin AF were expressed in arbitrary units (AU). With patients seated, all measurements were taken at the volar side of the lower arm, approximately 10–15 cm below the elbow. The levels of pentosidine, a major component of AGEs, were measured using skin biopsy at the volar side of the lower arm and appeared to correlate with skin AF [15]. The validity and reliability of skin AF levels in an Asian population measured using this method have been previously established [10, 14].

### Estimation of cardiovascular risk factors

Various clinical parameters, such as classic cardiovascular risk factors, left ventricular hypertrophy, insulin resistance, kidney function, brain natriuretic peptide (BNP) levels, oxidative stress, cardio-ankle vascular index as a marker of arterial stiffness, and hs-cTnT levels, were evaluated. The degree of obesity was estimated using body mass index, which was calculated as weight (kg) divided by height (m^2^). Current smoking was defined as smoking at least one cigarette per day during the previous 28 days. Right brachial blood pressure was measured twice using a mercury sphygmomanometer with participants in the sitting position. An average of two readings was used to determine systolic and diastolic blood pressures. Treatment with antihypertensive drugs was discontinued 24 h or even before prior to performing measurements. The severity of left ventricular hypertrophy was evaluated using Cornell (R wave in aVL + S wave in V3) electrocardiographic voltage calculations [16]. Diabetes mellitus was defined as a fasting blood glucose level ≥ 126 mg/dL or based on an ongoing treatment for diabetes. Dyslipidemia was defined as a low-density lipoprotein cholesterol level ≥ 140 mg/dL, a high-density lipoprotein cholesterol ≤40 mg/dL, a triglyceride level ≥ 150 mg/dL, or based on an ongoing treatment for dyslipidemia. This study used the cardio-ankle vascular index (CAVI) as a marker of arterial stiffness; CAVI was measured using a VaSera CAVI instrument (Fukuda Denshi Inc., Tokyo, Japan), following previously described methods [17]. Both brachial and ankle pulse waves were determined using inflatable cuffs; cuff pressure was maintained between 30 mmHg and 50 mmHg to ensure that it had only a minimal effect on systemic hemodynamics. The blood and pulse pressures were simultaneously determined with the subject in a supine position. CAVI was measured after the subjects rested for 10 min in a quiet room. The average coefficient of variation of CAVI has been shown to be <5%, which is small enough for clinical use and indicates that CAVI measurement has good reproducibility.

### Blood sampling

Blood samples were collected from the antecubital vein in the morning after 12 h of fasting. Glucose and insulin levels were measured using the glucose oxidase method and an enzyme immunoassay, respectively. To measure insulin resistance, homeostatic model assessment of insulin resistance (HOMA-IR) was calculated as follows [18]: HOMA-IR = fasting glucose concentration (mg/dL) × fasting insulin concentration (μg/mL)/405. Total cholesterol and triglyceride levels were measured using standard enzymatic methods. Serum high-density lipoprotein cholesterol levels were measured using selective inhibition. Serum low-density lipoprotein cholesterol levels were calculated using the Friedewald equation [19]. Participants with a serum triglyceride concentration ≥ 400 mg/dL were excluded because the method is accurate only below this concentration. The estimated glomerular filtration rate (eGFR) was calculated using the adjusted Modification of Diet in Renal Disease (MDRD) Study equation, proposed by the working group of the Japanese Chronic Kidney Disease Initiative [20]. Blood levels of BNP were measured using a commercial kit (SHIONOSPOT Reader; Shionogi & Co., Ltd., Osaka, Japan). The reactive oxygen metabolites (d-ROMs) test, which reflects blood hydroperoxide concentrations, was performed using a commercial kit (Diacron; Grosseto, Italy) [21]. Hs-cTnT levels were also measured using a commercial kit (Roche Diagnostics, Switzerland) [22]. For the hs-cTnT assay, the lower limit of detection was 0.003 ng/mL.

### Statistical analysis

A commercially available statistical software program (Stat View-J 5.0; HULINKS Inc., Tokyo, Japan) was used for all statistical analyses. Data are expressed as mean ± SD. Between-group comparisons were performed using the Student’s t test or Mann–Whitney U test, and the correlation coefficient was estimated using the Spearman rank-order correlation analysis. To clarify the independent factors that contributed to increased hs-cTnT levels, multiple regression analysis was performed using hs-cTnT as a subordinate factor. A *p*-value <0.05 was considered statistically significant.

## Results

**Fig. 1:**
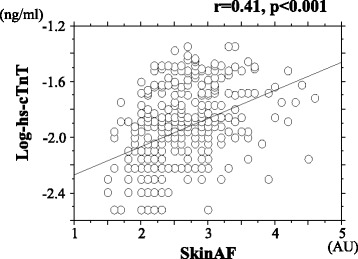
Correlations between Log-hs-cTnT and skin AF. A significantly positive correlation exists between Log-hs-cTnT and skin AF (*r* = 0.41, *p* < 0.001). r expressed correlation coefficient. hs-cTnT; high-sensitivity troponin T, AF; autofluorescence

Patient characteristics are shown in Table 1. Hs-cTnT was detected in 405 (88.6%) patients. Blood pressure levels demonstrated no significant differences between patients with detectable hs-cTnT and those with undetectable hs-cTnT. Age, body mass index, Cornell voltage, presence of diabetes mellitus, fasting blood glucose levels, Log-BNP levels, d-ROMs test, and CAVI were significantly higher, and eGFR was significantly lower, in patients with detectable hs-cTnT compared to those with undetectable hs-cTnT. Skin AF was significantly higher in patients with detectable hs-cTnT compared to those with undetectable hs-cTnT. Correlations between Log-hs-cTnT and skin AF are shown in Fig. 1. A significantly positive correlation was observed between these two parameters. Table 2 shows correlations between Log-hs-cTnT levels, skin AF, and various clinical parameters in the cohort with detectable hs-cTnT. Age, body mass index, Cornell voltage, presence of diabetes mellitus, eGFR, Log-BNP levels, d-ROMs test, and CAVI were all significantly correlated with Log-hs-cTnT levels. Sex, age, systolic blood pressure, presence of diabetes mellitus, fasting blood glucose levels, eGFR, Log-BNP levels, d-ROMs test, and CAVI were significantly correlated with skin AF.

**Table 1:** Patient characteristics

	Overall	hs-cTnT nondetection	hs-cTnT detection	*p* value
*n* (Male/Female)	457 (182/275)	52 (21/31)	405 (161/244)	0.953
Age (years)	67 ± 13	63 ± 12	68 ± 13	<0.01
Body mass index (Kg/m^2^)	22 ± 4	21 ± 3	23 ± 4	<0.05
Current smoker, *n* (%)	110 (24)	13 (25)	97 (24)	0.720
Systolic BP (mmHg)	158 ± 8	157 ± 10	158 ± 8	0.853
Diastolic BP x(mmHg)	92 ± 9	91 ± 9	92 ± 10	0.772
Pulse rate (mmHg)	68 ± 10	70 ± 11	68 ± 10	0.129
Cornell voltage (mm)	15 ± 5	12 ± 6	16 ± 5	<0.001
Diabetes mellitus, *n* (%)	174 (38)	18 (35)	156 (39)	<0.05
FBG (mg/dl)	113 ± 24	100 ± 20	118 ± 24	<0.05
IRI (μg/ml)	7.3 ± 4.4	7.1 ± 4.8	7.3 ± 4.4	0.062
HOMA-IR	2.1 ± 1.4	2.0 ± 1.3	2.2 ± 1.4	0.053
Dyslipidemia, *n* (%)	256 (56)	26 (50)	230 (57)	0.286
Total cholesterol (mg/dl)	221 ± 42	218 ± 40	221 ± 41	0.341
LDL cholesterol (mg/dl)	142 ± 37	140 ± 39	142 ± 38	0.358
Triglyceride (mg/dl)	148 ± 70	146 ± 83	149 ± 69	0.279
HDL cholesterol (mg/dl)	49 ± 16	49 ± 15	49 ± 16	0.820
eGFR (ml/min/1.73m^2^)	64 ± 21	69 ± 21	63 ± 18	<0.05
Log-BNP (pg/ml)	1.8 ± 0.4	1.6 ± 0.4	1.8 ± 0.4	<0.01
d-ROMs test (U. Carr)	333 ± 102	297 ± 107	338 ± 101	<0.001
CAVI	8.9 ± 1.4	8.4 ± 1.2	9.0 ± 1.4	<0.001
Skin AF	2.6 ± 0.5	2.2 ± 0.5	2.6 ± 0.5	<0.001
Log-hs-cTnT (ng/ml)	−1.9 ± 0.3	–	−1.9 ± 0.3	–
Medication				
CCB, *n* (%)	338 (74)	38 (73)	300 (75)	0.712
RAS inhibitor, *n* (%)	229 (50)	25 (48)	207 (51)	0.653
Statin, *n* (%)	183 (40)	20 (38)	163 (41)	0.699

Data are expressed mean ± SD, *hs-cTnT* high-sensitivity cardiac troponin T, *BP* blood pressure, *FBG* fasting blood glucose, *IRI* immuno reactive insulin, *HOMA-IR* homeostatic model assessment of insulin resistance, *LDL* low-density lipoprotein, *HDL* high-density lipoprotein, *eGFR* estimated glomerular filtration rate, *BNP* brain natriuretic peptide, *d-ROMs* derivatives of reactive oxygen metabolites, *CAVI* cardio- ankle vascular index, *AF* autofuluorescence, *CCB* calcium channel blocker, *RAS* renin- angiotensin system

**Table 2:** Correlation between Log-hs-cTnT, Skin AF and clinical parameters in hs-cTnT detectable patients

	Log-hs-cTnT	Skin AF
	r	r
Sex (Female = 0, Male = 1)	0.05	0.13**
Age	0.33*	0.25*
Body mass index	0.14**	0.08
Current smoker (No = 0, Yes = 1)	0.03	0.09
Systolic BP	0.08	0.10***
Diastolic BP	0.06	0.08
Pulse rate	0.05	0.06
Cornell voltage	0.22*	0.10
Diabetes mellitus (No = 0, Yes = 1)	0.12**	0.34*
FBG	0.06	0.14**
IRI	0.05	0.07
Log-HOMA-IR	0.06	0.10
Dyslipidemia (No = 0, Yes = 1)	−0.03	−0.01
Total cholesterol	−0.05	0.04
LDL cholesterol	−0.06	0.04
Triglyceride	−0.03	0.07
HDL cholesterol	−0.07	−0.05
eGFR	−0.15**	−0.20*
Log-BNP	0.25*	0.11***
d-ROMs test	0.41*	0.40*
CAVI	0.41*	0.42*
CCB (No = 0, Yes = 1)	−0.05	−0.06
RAS inhibitor (No = 0, Yes = 1)	−0.08	−0.07
Statin (No = 0, Yes = 1)	−0.07	−0.08

r expressed correlation coefficient

*p < 0.001, **p < 0.01, ***p < 0.05. Abbreviations as in Table 1

The results of multiple regression analysis are presented in Table 3. Selections of explanatory factors were decided by check of multicollinearity among variables or performance of stepwise method. Skin AF, CAVI, d-ROMs test, Cornell voltage, Log-BNP levels, age, and eGFR were selected as independent variables for Log-hs-cTnT.

**Table 3:** Multiple regression analysis for Log-hs-cTnT

Explanatory factor	β	95% CI	*p* value
Skin AF	0.30	0.096–0.214	<0.001
CAVI	0.23	0.021–0.059	<0.001
d-ROMs test	0.20	0.001–0.002	<0.001
Cornell voltage	0.19	0.002–0.011	<0.001
Log-BNP	0.16	0.039–0.152	<0.01
Age	0.16	0.001–0.005	<0.01
eGFR	−0.15	−0.003 − −0.001	<0.01
Body mass index	0.09	−0.001 − 0.025	0.072
	R^2^=0.30,	*F* value = 23.9,	*p* < 0.001

95% CI are expressed as 95%CI of partial regression coefficient. *hs-cTnT* high-sensitivity cardiac troponin T, *β* standardized regression coefficient, *CI* confidence interval, *AF* autofluorescence, *CAVI* cardio-ankle vascular index, *d-ROMs* derivatives of reactive oxygen metabolites, *BNP* brain natriuretic peptide, *eGFR* estimated glomerular infiltration rate

## Discussion

This study shows a significant relationship between hs-cTnT and skin AF, reflecting tissue accumulation of AGEs at the volar side of the lower arm. Measurement of skin AF is very simple and reliable; therefore, the results of this study indicate that skin AF is a useful biomarker to predict the degree of subclinical myocardial damage in hypertensive patients.

A clinical study of a healthy population [4] revealed detectable hs-cTnT in 67.7% of the population, with a detection limit of 0.003 ng/mL. On the contrary, Satoh et al. reported that hs-cTnT was also detected in 184 (78.0%) patients among 236 hypertensive patients using an identical detection limit as above [5]. The results of the present study also indicated detectable hs-cTnT in 88.6% of the population using the same detection limit as above. Thus, the results of the present study and previous reports indicate that hs-cTnT is highly detected in hypertensive patients, suggesting that myocardial injury in hypertensive patients is progressive during the subclinical stages of heart failure. Sato et al. also reported that age, eGFR, and Cornell voltage were significantly associated with hs-cTnT based on multivariate analysis. In addition, other clinical reports indicated a significant relationship between BNP and hs-cTnT [1, 23]. The analysis in this study also selected these factors as independent variables for hs-cTnT as a subordinate factor.

Hofmann et al. examined the relationship between AGE-modified cardiac tissue collagen levels and skin AF and found a significant relationship between cardiac tissue glycation and skin AF [24]. Their report indicated that the levels of AGEs found at the volar side of the lower arm appear to reflect the degree of AGE accumulation in cardiomyocytes. In addition, other studies have indicated several pathways by which AGEs or receptors of AGEs (RAGEs) could influence myocardial injury in a diabetes model. Ma et al. [25] reported that diabetes resulted in a significant increase in AGE and RAGE levels in the heart, especially in cardiomyocytes, using mice with streptozotocin-induced diabetes. These authors also reported that AGE-induced cardiomyocyte dysfunction was linked to mitochondrial membrane depolarization and reduced GSK-3β inactivation, events that can be prevented by RNA interference knockdown of RAGE expression. Brett et al. [26] also reported that RAGE is present in cardiomyocytes and suggested the potential relevance of AGE–RAGE interactions for modulating cardiac function in diabetes. Thus, AGEs and RAGEs are believed to play important roles in myocardial injury under diabetic conditions. Moreover, in this study, skin AF was selected as a strong independent variable with Log-hs-cTnT as a subordinate factor. Therefore, AGEs and RAGEs possibly affect myocardial injury in hypertensive patients.

Oxidative stress is closely associated with heart failure progression or vascular events. Several pathways whereby oxidative stress leads to myocardial injury have been identified, including dysfunction of the mitochondrial electron transport complex, nicotinamide adenine dinucleotide phosphate oxidase activity, and myocardial cell apoptosis [27, 28]. The present study reveals the importance of oxidative stress in myocardial injury occurring with hypertension and during the subclinical stages of heart failure. In addition, a number of basic and clinical studies have shown a close association between AGEs or RAGEs and oxidative stress. An association between AGEs or RAGEs and oxidative stress in myocardial cells has been reported [29]. In this study, a significant correlation was observed between skin AF and a positive d-ROMs test as a marker of oxidative stress in vivo, suggesting that the association between AGEs or RAGEs and oxidative stress in myocardial cells is causative of hs-cTnT elevation in hypertensive patients.

The principle of CAVI is known to be a marker of arterial stiffness, which is independently associated with blood pressure levels [17]. In recent years, several studies have indicated the importance of left ventricular dysfunction in the progression of myocardial injury; furthermore, increases in aortic artery stiffness are known to be caused by left ventricular dysfunction [30]. On the contrary, skin AF had a significantly positive correlation with CAVI in this study. Other studies have also indicated a significant relationship between skin AF and arterial stiffness [12, 31]. In addition, basic studies have reported that RAGE is expressed in vascular tissues such as endothelium, smooth muscle cells, and mononuclear cells [26]. Therefore, the relationship between hs-cTnT and CAVI may be partly explained by the eventual consequence of myocardial injury via left ventricular dysfunction, resulting from an increase in vascular resistance or afterload caused by the accumulation of AGEs in arterial tissues.

This study has several limitations. First, angiography, computed tomography, and magnetic resonance imaging were not performed. Therefore, asymptomatic cardiovascular diseases may have gone undetected. Second, medical treatments including the administration of anti-hypertensive drugs or statins may have influenced the study results, although the study results indicate that the effects of these drugs were insignificant. Finally, this study was cross-sectional in a single unit, and the sample size was relatively small. Additional prospective studies including evaluations of interventional therapies are required to clarify the clinical significance of skin AF as a risk factor for heart failure or vascular events in hypertensive patients.

## Conclusions

In conclusion, the findings of this study indicate that skin AF is an important determining factor for hs-cTnT elevation in hypertensive patients with no history of cardiovascular events.

## Abbreviations

AF: Autofluorescence
AGEs: Advanced glycation endoproducts
BNP: Brain natriuretic peptide
BP: Blood pressure
CAVI: Cardio-ankle vascular index
CCB: Calcium channel blocker
CI: Confidence interval
eGFR: Estimated glomerular filtration rate
FBG: Fasting blood glucose
HDL: High-density lipoprotein
HOMA-IR: Homeostasis model assessment as of insulin resistance
Hs-cTnT: High-sensitivity cardiac troponin T
IRI: Immuno reactive insulin
LDL: Low-density lipoprotein
RAS: Renin- angiotensin system
SD: Standard deviation
